# INDIVIDUAL DIFFERENCES IN TIME OF DAY EFFECTS ON AMBULATORY COGNITIVE FUNCTIONING IN MIDDLE AND OLDER ADULTS

**DOI:** 10.1093/geroni/igae098.2213

**Published:** 2024-12-31

**Authors:** Emorie Beck, Zoe Hawks, Laura Germine

**Affiliations:** University of California Davis, Davis, California, United States; McLean Hospital/Harvard Medical School, Belmont, Massachusetts, United States; McLean Hospital/Harvard Medical School, Belmont, Massachusetts, United States

## Abstract

Previous work indicates that (1) changes in diurnal cycles are associated with the onset and progression of dementia and cognitive impairment and (2) that cognitive function itself tends to show diurnal cycles in the laboratory. But no work to date has examined whether cognitive function assessed via ecological momentary assessment can recover such daily cycles and whether individual differences in those cycles are associated with short- and long-term outcomes. In this presentation, we examine whether ambulatory cognitive assessments assessed via ecological momentary assessment (EMA) demonstrate intra-day cycles of cognitive performance. This study specifically aims to test whether such cycles emerge, whether there are individual differences in those cycles, and whether individual differences are associated with cross-sectional measures of cognitive health and psychosocial risk factors. At baseline, 200 (Mean age = 55; 50.1% female) participants responded to five surveys per day for 20 days capturing their cognitive function, contextual factors, and psychological experiences. We will examine nonlinear changes in cognitive function through the day using both multilevel time series decomposition and cosinor models, which also allow us to examine individual differences in these patterns. We will then examine whether these individual differences are concurrently associated with global cognitive functioning, cognitive impairment, personality traits, and other psychosocial factors.

